# Sodium channel activation underlies transfluthrin repellency in *Aedes aegypti*

**DOI:** 10.1371/journal.pntd.0009546

**Published:** 2021-07-08

**Authors:** Felipe Andreazza, Wilson R. Valbon, Qiang Wang, Feng Liu, Peng Xu, Elizabeth Bandason, Mengli Chen, Shaoying Wu, Leticia B. Smith, Jeffrey G. Scott, Youfa Jiang, Dingxin Jiang, Aijun Zhang, Eugenio E. Oliveira, Ke Dong

**Affiliations:** 1 Department of Entomology, Michigan State University, East Lansing, Michigan, United States of America; 2 Department of Entomology, Universidade Federal de Viçosa, Viçosa, Brazil; 3 Department of Biology, Duke University, Durham, North Carolina, United States of America; 4 Institute of Pesticide and Environmental Toxicology, Zhejiang University, Hangzhou, China; 5 Department of Entomology, Cornell University, Ithaca, New York, United States of America; 6 Jiangsu Yangnong Chemical Co., Ltd., Jiangsu, China; 7 Key Laboratory of Natural Pesticide and Chemical Biology, Ministry of Education, South China Agricultural University, Guangzhou, China; 8 Invasive Insect Biocontrol and Behavior Laboratory, Beltsville Agricultural Research Center-West, USDA-ARS, Beltsville, Maryland, United States of America; Universita degli Studi di Pavia, ITALY

## Abstract

**Background:**

Volatile pyrethroid insecticides, such as transfluthrin, have received increasing attention for their potent repellent activities in recent years for controlling human disease vectors. It has been long understood that pyrethroids kill insects by promoting activation and inhibiting inactivation of voltage-gated sodium channels. However, the mechanism of pyrethroid repellency remains poorly understood and controversial.

**Methodology/Principal findings:**

Here, we show that transfluthrin repels *Aedes aegypti* in a hand-in-cage assay at nonlethal concentrations as low as 1 ppm. Contrary to a previous report, transfluthrin does not elicit any electroantennogram (EAG) responses, indicating that it does not activate olfactory receptor neurons (ORNs). The *1S*-*cis* isomer of transfluthrin, which does not activate sodium channels, does not elicit repellency. Mutations in the sodium channel gene that reduce the potency of transfluthrin on sodium channels decrease transfluthrin repellency but do not affect repellency by DEET. Furthermore, transfluthrin enhances DEET repellency.

**Conclusions/Significance:**

These results provide a surprising example that sodium channel activation alone is sufficient to potently repel mosquitoes. Our findings of sodium channel activation as the principal mechanism of transfluthrin repellency and potentiation of DEET repellency have broad implications in future development of a new generation of dual-target repellent formulations to more effectively repel a variety of human disease vectors.

## Author summary

Vector-transmitted human diseases, such as dengue fever, represent serious global health burdens. Pyrethroids, including transfluthrin, are widely used as insecticides and repellents due to their low mammalian toxicity and relatively benign environmental impact. Pyrethroids target voltage-gated sodium channels for their insecticidal action. However, the mechanism of pyrethroid repellency remains unclear and controversial. Insect repellency is traditionally thought to be mediated by olfactory receptors. We made two important discoveries in this study, showing that transfluthrin repellency is *via* activation of sodium channels and transfluthrin enhances DEET repellency. Discovery of sodium channel activation as a major mechanism of pyrethroid repellency has broad significance in insect olfaction study, repellents development, and control of human disease vectors.

## Introduction

Pyrethroid insecticides are synthetic analogues of natural pyrethrins, which are major insecticidal components of the pyrethrum extract from the flowers of *Chrysanthemum* species [1]. The insecticidal activity of pyrethroids is through their action on voltage-gated sodium channels, which are critical for electrical signaling in the nervous system. Pyrethroids promote activation and inhibit inactivation of sodium channels, resulting in repetitive firing and/or membrane depolarization and eventual nerve conduction block and paralysis (i.e., knockdown) [2,3]. Mutations in the sodium channel confer a major type of pyrethroid resistance, known as knockdown resistance (kdr) [3–5].

Pyrethroids have been used extensively in the control of vectors of human diseases, including malaria and dengue. Volatile pyrethroids are also used as popular repellents globally, in the form of vaporizers, emanators, mats, and coils, against mosquitoes [6–15]. For example, as one of the most widely used volatile pyrethroids, transfluthrin has been incorporated into a variety of new mosquito control products and programs. When mosquito coils treated with 0.03% transfluthrin were used either alone or in combination with long-lasting insecticide bed nets (LLINs), malaria parasite prevalence was reduced by up to 94% [10]. Application of transfluthrin to hessian or sisal decorations/products, eave ribbons, eave-baffles and window screens offers a promising method of mosquito contact/bit prevention in both indoor or outdoor settings [11,13,14,16–20].

Despite the importance of pyrethroid-treated products in preventing mosquito biting and reducing disease transmission, and laboratory behavioral assays showing transfluthrin-mediated spatial (i.e., non-contact) repellency in *Ae*. *aegypti* mosquitoes [21–23], the mechanistic basis of pyrethroid spatial repellency remains poorly understood and controversial. For example, an earlier study showed that a pyrethroid (TL-I-73) inhibits the activities of odorant receptors (Ors) induced by volatiles indole and R-(-)-1-octen-3-ol in *Xenopus* oocytes, suggesting that the repellent activity of pyrethroids may be accounted for by their inhibitory effects on Ors [24]. Another study showed that transfluthrin repellency was reduced in pyrethroid-resistant *Ae*. *aegypti* mosquitoes carrying a kdr mutation [21] and proposed that neurotoxic irritation of mosquitoes by sublethal doses of transfluthrin as a mechanism of transfluthrin repellency [21]. A more recent study [25] also showed reduced repellency by transfluthrin and metofluthrin in another pyrethroid-resistant *Ae*. *aegypti* strain, Puerto Rico, carrying multiple kdr mutations (V410L, V1016I, F1534C) [26,27]. However, surprisingly, these Puerto Rico mosquitoes also exhibited resistance to repellency by mosquito repellents DEET, 2-undecanone and IR3535, which do not act on sodium channels. Furthermore, the same study [25] showed that transfluthrin and metofluthrin elicited electroantennogram (EAG) responses (due to activation of olfactory receptor neurons in mosquito antennae) in adults of *Ae*. *aegypti* mosquitoes and the amplitudes of EAG signals elicited by pyrethroids and non-pyrethroid repellents were reduced in kdr mosquitoes. These authors [25] suggested that the reduced sensitivity of Puerto Rico mosquitoes to pyrethroids and non-pyrethroid repellents may represent a general fitness cost associated with the kdr mutations in Puerto Rico mosquitoes. Accordingly, the fundamental questions of (i) whether pyrethroids activate or inhibit Ors activities and (ii) whether activation of sodium channels *per se* mediates transfluthrin repellency remain unclear.

In this study we took a combination of molecular genetics, electrophysiological and behavioral approaches to understand the mechanism of transfluthrin repellency. Our results show that sodium channel activation alone, not activation of olfactory receptors, is a principal mechanism of repelling insects and transfluthrin enhances Or-mediated DEET repellency. Our findings have significant practical implications for understanding the modes of action of pyrethroids as insect repellents as well as for the development of a new generation of synergistic repellent mixtures that may more effectively combat mosquitoes and other human disease vectors.

## Methods

### Insects and chemicals

Five *Ae*. *aegypti* mosquito lines were used: Two wild-type lines, Rockefeller and Orlando (BEI Resources, NIAID, NIH); two pyrethroid-resistant lines KDR:ROCK [28], and Puerto Rico line (BEI Resources, NIAID, NIH); and the olfactory defective *orco*^*-/-*^ line (*orco*^*16*^) (BEI Resources, NIAID, NIH) [29]. All the odorants used in this study and its sources are provided in S1 Table.

### Hand-in-cage assay

**Fig 1:**
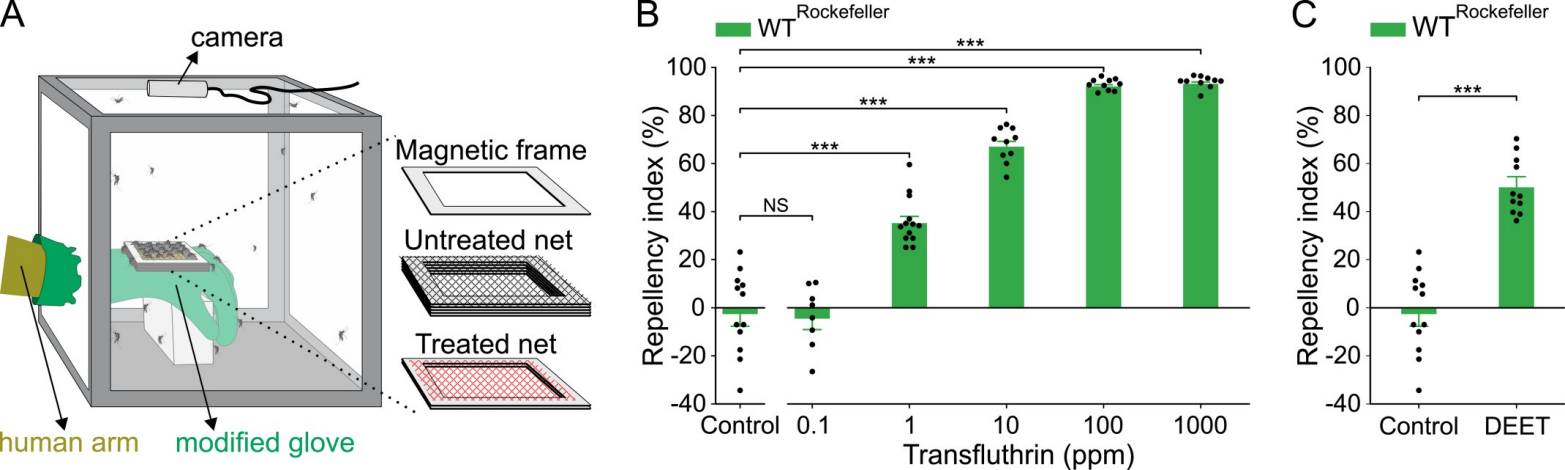
Transfluthrin elicits spatial repellency in *Ae*. *aegypti*. (A) A schematic drawing of the hand-in-cage setup. (B) Dose-dependence of transfluthrin repellency in Rockefeller; Student’s *t*-test, *t* = 0.25, *df* = 18, *P* = 0.802 for 0.1 ppm, Mann-Whitney Rank Sum test, *U* = 0, *P* < 0.001 for both 1, 10, 100 and 1000 ppm; NS = not significant, ***P < 0.001; *n* values for: control = 12, 0.1 ppm = 8, 1 ppm = 13, and the rest = 10. (C) Repellency by DEET (1 ppm) in Rockefeller; Student’s *t*-test, *t* = 8.45, *df* = 21, *P* < 0.001; ***P < 0.001; *n* values for: control = 12 and DEET = 11. The control represents the baseline activity in response to the solvent. Data are presented as mean ± SEM. Dots over the bars represent individual replicate values.

The hand-in-cage behavioral assay followed similar procedures from Boyle et al [30]. Briefly, a group of four to nine days-old females (about 40, mated, non-blood fed) inside a mosquito cage (30 cm x 30 cm x 30 cm) (BioQuip, Rancho Dominguea, CA) were exposed to a human hand wearing a modified glove with a window covered with two pieces of netting, as detailed in Fig 1A. The bottom netting (5.5 cm x 6.5 cm) was treated with 500 μl of either solvent (acetone), or test compounds. The top netting was not treated and prevented mosquitoes from contacting the treated netting and the hand. A digital camera (e-con Systems Inc, San Jose, CA, model: e-CAM51A) on the top of the cages recorded mosquito landing on the top netting for five minutes. For each hand-in-cage experiment, 10 ± 2 cages were tested with acetone-treated netting first, then in the same sequence, the ten cages were tested using test compound-treated netting (i.e., treatment). The time interval of assays between the first trial (solvent) and second trial (treatment) was at least 1.5 hours. Controls were from two trials of solvent 1.5 hours apart to make sure that mosquitoes continue landing in the second trial at the same rate. Repellency index was calculated using the following equation: Percentage repellency = [1 - (cumulative number of landings on the window of treatment / cumulative number of landings on the window of solvent treatment)] x 100). The assay was run at 27–30°C and relative humidity of 30–50% by at least two different hosts (i.e., testers) for each experiment.

### Electrophysiology recordings

Electroantennogram were performed as described elsewhere [31,32] using a recording system by Syntech company (Kirchzarten, Germany), which consisted of universal electrode holders, a preamplifier (10x), an analog-to-digital signal converter (IDAC-4) and a stimulus controller (CS-55). The software EAGPro (Syntech) were used to visualize, record and analyze the data. Insect antennae were bathed in a humidified air flow (1.2 l/min) under a microscope (100–1000x, Nikon Eclipse FN1, Japan), and odorants were delivered by air flow (0.5 ml/min) into a glass Pasteur pipet serving as an odorant cartridge. For delivery, 1 μl of pure odorants or transfluthrin was applied at the inner wall of the glass cartridge.

To evaluate the activities of transfluthrin and its inactive *1S-cis* isomer on mosquito wild-type and mutant sodium channels, the wild-type AaNa_v_1-1 and AaNa_v_1-1 mutant carrying two kdr mutations (S989P and V1016G) were expressed in *Xenopus* oocytes system and functionally characterized using two-electrode voltage clamp as previously described [33,34]. Any relevant detail is further denoted in the appropriate figure legend or text.

### Data analysis and statistics

SigmaPlot 12.5 (Systat Software) was used to perform the statistics analysis and plot the figures. After being plotted, figures were further edited (for color, labelling, etc.) and assembled in CorelDRAW Graphic Suit 2020—version 22 (Corel Corporation, Ottawa, Canada). Unpaired Student’s *t*-test was used to compare two sets of data. If data did not meet the normality or equality of the variance assumptions needed for Student’s *t*-test, the equivalent Mann-Whitney Rank Sum test was used instead. For paired comparison of multiple treatments on a same individual against control a Friedman RM ANOVA on Ranks was used, with Dunnett’s multiple comparison against a control treatment.

## Results

### Transfluthrin elicits spatial (i.e., non-contact) repellency in *Ae*. *aegypti* in a hand-in-cage assay

To evaluate spatial repellency by transfluthrin in the presence of an attraction, a human hand, we adopted a hand-in-cage assay developed by Boyle et al [30]. This assay setup involves placement of a human hand with a modified glove in a mosquito cage (Fig 1A). When mosquitoes in the cage are attracted to the human hand, the top netting serves as physical barrier preventing mosquitoes from direct contact with the bottom netting (above the hand) and the hand. When the bottom netting was treated with acetone as control, the landing of mosquitoes on the top netting was not affected. However, when the bottom netting was treated with transfluthrin, significantly less frequent landing of mosquitoes was observed (Fig 1B). This repellent effect by transfluthrin vapor was observed at concentrations as low as 1 ppm of transfluthrin (i.e., 20 ng/cm^2^) in Rockefeller (wild-type) mosquitoes (Fig 1B). Similarly, when the bottom netting was treated with DEET (at 1 ppm), a gold-standard mosquito repellent, significant less frequent landing of mosquitoes was observed (Fig 1C). Greater repellency was observed at higher concentrations of transfluthrin (Fig 1B). However, at 100 ppm, about 10% of the mosquitoes exhibited uncoordinated locomotion, and at 1000 ppm, a small fraction of mosquitoes (8.42 ± 2.2%) were knocked down during the assay. No such locomotive modifications were observed at the lower concentrations. In the early stage of running the hand-in-cage assay using transfluthrin, we continued monitor mosquito behavior in the cages every ten minutes over a period of one hour, and the mortality over a period of 24h after the hand-in-cage assay was done (i.e., after the hand with transfluthrin was removed). During this post-assay period, we did not observe any abnormal behavior and mortality of the mosquitoes in the cages that have been exposed to transfluthrin at 1 ppm or lower in the hand-in-cage assay. Therefore, in subsequent experiments, we evaluated transfluthrin repellency only at 1 ppm or below to avoid complications from the insecticidal and/or neurotoxic activity of transfluthrin.

### Transfluthrin does not evoke any electroantennogram (EAG) signal

**Fig 2:**
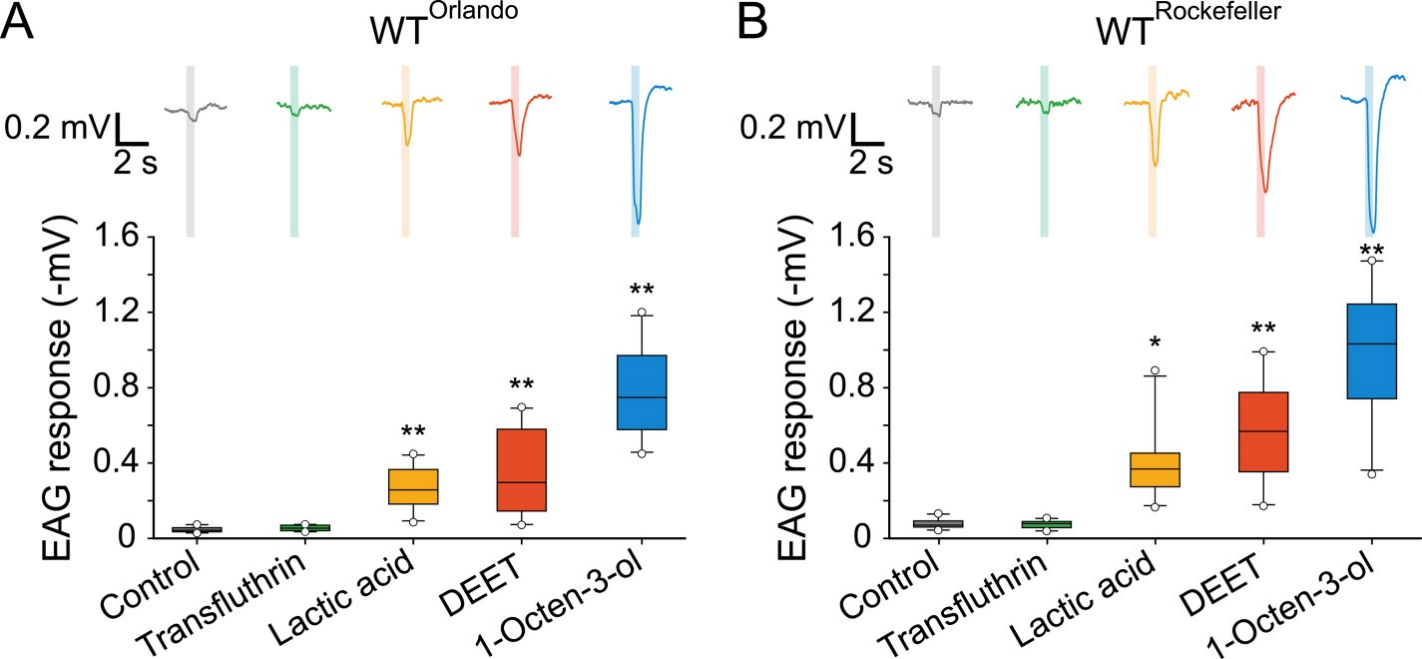
Transfluthrin does not elicit electroantennogram in *Ae*. *aegypti*. Electroantennogram performed with two wild-type lines, Orlando (A) and Rockefeller (B). A representative trace is shown above each plot; the asterisks (*P < 0.05, **P < 0.01) indicate significant differences against control within each mosquito line, Friedman RM ANOVA on Ranks, χ^2^ = 46.49, *df* = 5, *P* < 0.001 for Orlando (A), and χ^2^ = 43.83, *df* = 5, *P* < 0.001 for Rockefeller (B); *n* = 10 antennae. Boxes represents the 25^th^, 50^th^ and 75^th^, whiskers the 10^th^ and 90^th^, and white circles the 5^th^ and 95^th^ percentiles of the data.

Yang and colleagues [25] recently reported that transfluthrin induces EAG responses in *Ae*. *aegypti*, including Orlando (wild-type) and Puerto Rico (pyrethroid-resistant) mosquitoes. During the early stage of this project, we also found that some transfluthrin samples, provided by a former collaborator, elicited EAG responses in *Ae*. *aegypti*. However, we subsequently found those transfluthrin samples contained impurities. When we repeated the experiment using transfluthrin from Sigma-Aldrich (99.2–99.9% purity), we could not detect any EAG signals in either Rockefeller, Orlando or Puerto Rico mosquitoes, even when undiluted transfluthrin was delivered (Figs 2 and S1). Similarly, no EAG responses were detected by transfluthrin from Jiangsu Yangnong Chemical Co. Ltd. (Jiangsu, China; 98.5% purity) (S1 Fig). In contrast (and as expected), DEET, 1-octen-3-ol and lactic acid all evoked EAG responses (Figs 2 and S1). We speculate that impurities in our earlier transfluthrin samples were responsible for eliciting EAG signals in our initial experiments.

### Transfluthrin repellency is not reduced in *orco*^*-/-*^ mosquitoes

**Fig 3:**
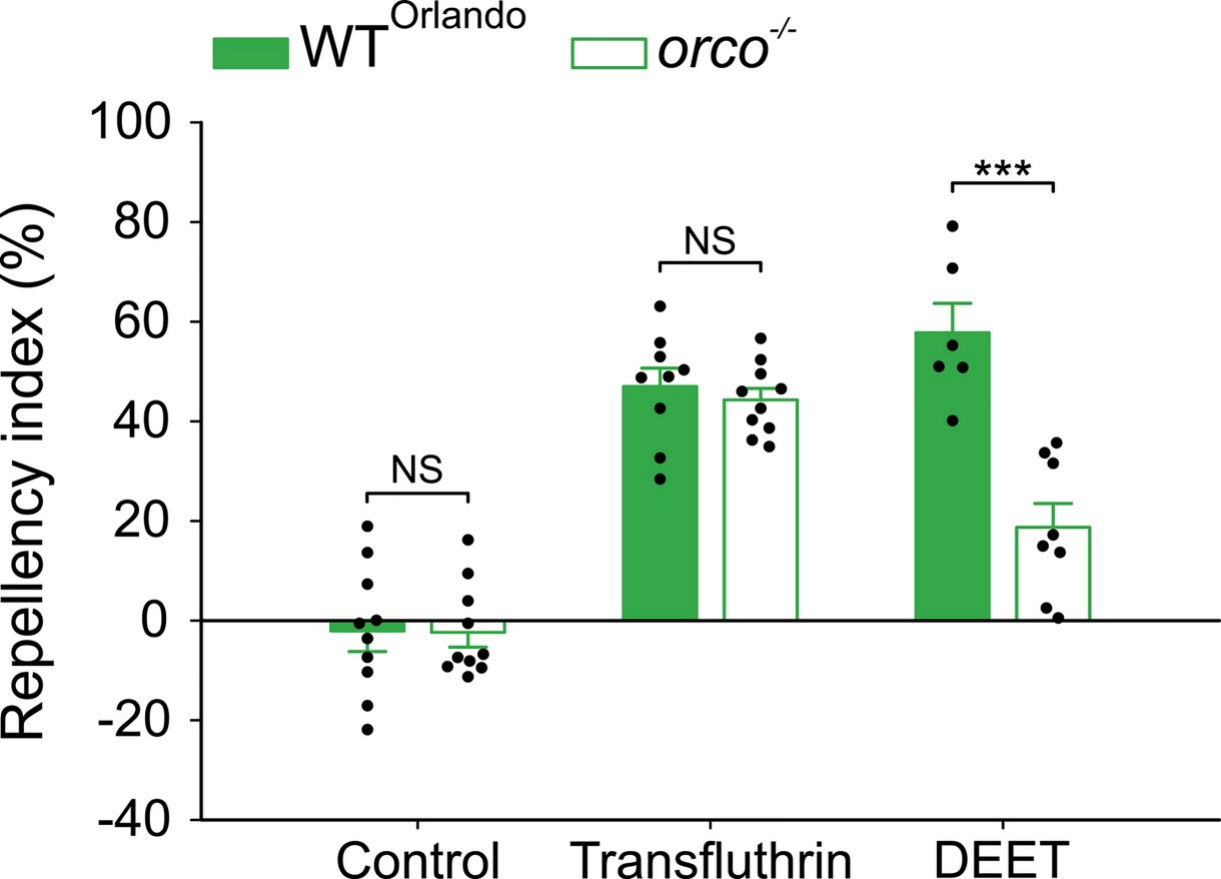
Transfluthrin repellency does not requires activation of Ors in *Ae*. *aegypti*. Repellency by 1 ppm of both transfluthrin and DEET in Orlando and *orco*^*-/-*^ mosquitoes; Student’s *t*-test, control: *t* = 0.05, *df* = 18, *P* = 0.963, transfluthrin: *t* = 0.64, *df* = 17, *P* = 0.530, DEET: *t* = 5.17, *df* = 12, *P* < 0.001, NS = not significant, ***P < 0.001; *n* values for: both controls and transfluthrin in *orco*^*-/-*^ = 10, transfluthrin in Orlando = 9, DEET in Orlando = 6, and DEET in *orco*^*-/-*^ = 8. The controls represent the baseline activity in response to the solvent; Data are presented as mean ± SEM. Dots over the bars represent individual replicate values.

Next, we evaluated transfluthrin and DEET repellency in an *Ae*. *aegypti* mutant, *orco*^*-/-*^, in which the odorant receptor co-receptor gene (*orco*) was mutated resulting in impaired Or-mediated olfactory pathways because Orco is essential for the function of Ors [29]. DEET repellency was reduced in *orco*^*-/-*^ mosquitoes (Fig 3) compared to the wild-type *Ae*. *aegypti* strain Orlando, from which the *orco*^*-/-*^ mutant was generated [29], consistent with the observation that DEET non-contact repellency is Or/Orco-dependent [29]. However, transfluthrin repellency was not reduced in *orco*^*-/-*^ mosquitoes (Fig 3), further indicating that transfluthrin repellency is Or/Orco-independent (i.e., does not require activation of Or/Orco). Collectively, results from EAG and the *orco*^*-/-*^ mutant contradict the notion that transfluthrin activates ORNs as part of its repellent mechanism.

### Transfluthrin repellency depends on activation of voltage-gated sodium channels

**Fig 4:**
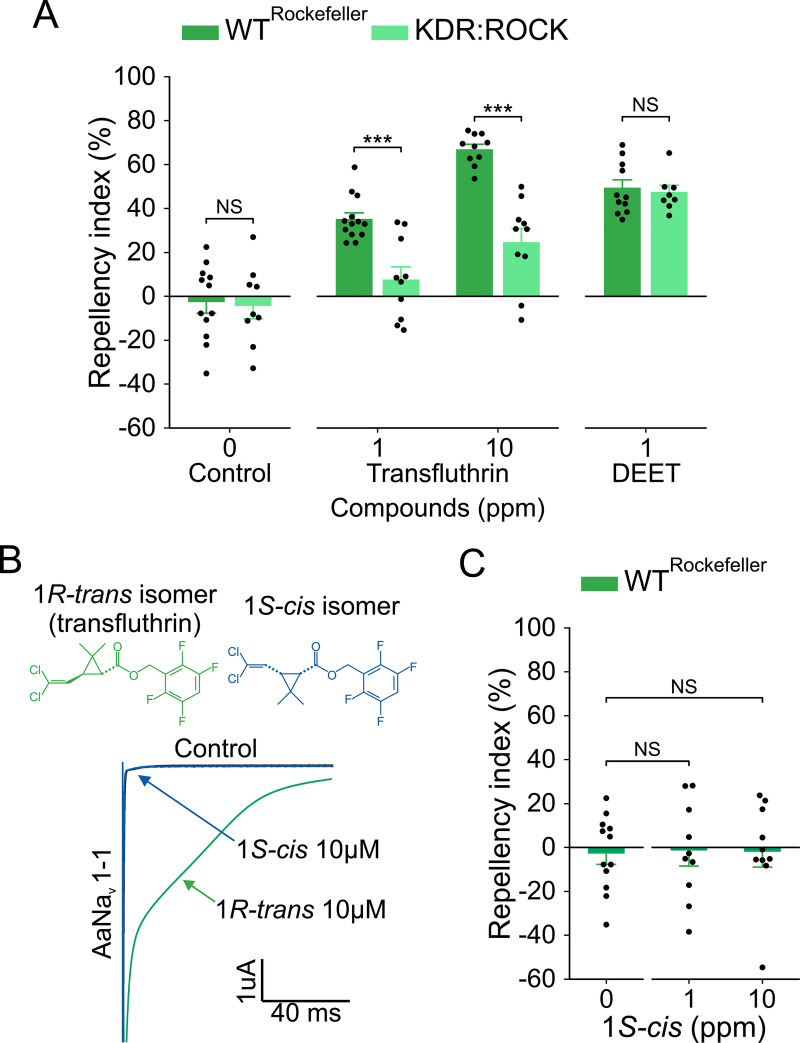
Transfluthrin repellency depends on its action on voltage-gated sodium channels. (A) Repellency by transfluthrin and DEET in Rockefeller and KDR:ROCK mosquitoes. Student’s *t*-test, for control: *t* = 0.20, *df* = 19, *P* = 0.840, for transfluthrin 1 ppm: *t* = 4.62, *df* = 21, *P* < 0.001, for transfluthrin 10 ppm: *t* = 6.39, *df* = 18, *P* < 0.001, for DEET: *t* = 0.39, *df* = 17, *P* = 0.701, NS = not significant, ***P < 0.001; *n* values for: KDR:ROCK for DEET = 8, KDR:ROCK for control = 9, Rockefeller for DEET = 11, Rockefeller for control = 12, Rockefeller for transfluthrin 1 ppm = 13, and the rest = 10. (B) Measurement of tail-current induced by *1R-trans* (transfluthrin) or *1S-cis* isomers following a 100-pulse train of 5-ms depolarization from -120 mV to 0 mV with 5-ms interval from AaNa_v_1-1 channels in *Xenopus* oocytes [35]. (C) Repellency by *1S-cis* isomer in Rockefeller; Student’s *t*-test, for 0 *vs* 1 ppm: *t* = 0.1, *df* = 20, *P* = 0.922, for 0 *vs* 10 ppm: *t* = 0.16, *df* = 20, *P* = 0.871, NS = not significant; *n* values for: control = 12, and the rest = 10. The controls represent the baseline activity in response to the solvent. Data in panels A and C are presented as mean ± SEM. Dots over the bars represent individual replicate values.

To determine the involvement of sodium channel activation in transfluthrin repellency, we used Rockefeller (wild-type) and KDR:ROCK (pyrethroid-resistant, carrying the S989P+V1016G *kdr* allele, as the only mechanism of pyrethroid resistance within the genetic background of Rockefeller (also called ROCK) [28]) strains in the hand-in-cage assay. KDR:ROCK mosquitoes were 30-fold more resistant to knockdown by transfluthrin than Rockefeller mosquitoes in a vapor toxicity bioassay (S2 Fig). We also confirmed that AaNa_v_1-1 channels carrying the two kdr mutations were resistant to transfluthrin compared to AaNa_v_1-1 wild-type channels (S2 Fig), similar to earlier studies reporting that the double mutation channel was resistant to permethrin and deltamethrin, two pyrethroids [35,36]. As shown in Fig 4A, transfluthrin repellency was reduced in KDR:ROCK compared to Rockefeller. Furthermore, we found that repellency by DEET was similar in Rockefeller and KDR:ROCK mosquitoes (Fig 4A), indicating that S989P+V1016G mutations did not reduce DEET repellency.

To further evaluate the role of sodium channels in transfluthrin repellency, we took advantage of stereospecific effects of pyrethroids on sodium channels and toxicity [3,37]. Pyrethroids possess alternative chiral configurations at C1 and C3 of the cyclopropane ring. *1R*-*cis* and *1R*-*trans* isomers are active, whereas *1S*-*cis*, and *1S*-*trans* isomers are inactive [3,37]. Commercial transfluthrin is in *1R*-*trans* configuration (Fig 4B). Upon repolarization under voltage-clamp conditions, transfluthrin induced tail-currents in the *Ae aegypti* sodium channel, AaNa_v_1-1, expressed in *Xenopus* oocytes (Fig 4B), indicating the prolonged opening of sodium channels by transfluthrin. In contrast, *1S*-*cis* isomer did not induce any tail current (Fig 4B), indicating that *1S*-*cis* isomer cannot act on sodium channels. As expected, *1S*-*cis* isomer did not induce knockdown in a vapor toxicity bioassay (S2 Fig). Importantly, *1S*-*cis* isomer did not elicit spatial repellency (Fig 4C). These results clearly showed that activation of sodium channels is essential for transfluthrin to elicit repellency.

### Transfluthrin enhances the potency of DEET

**Fig 5:**
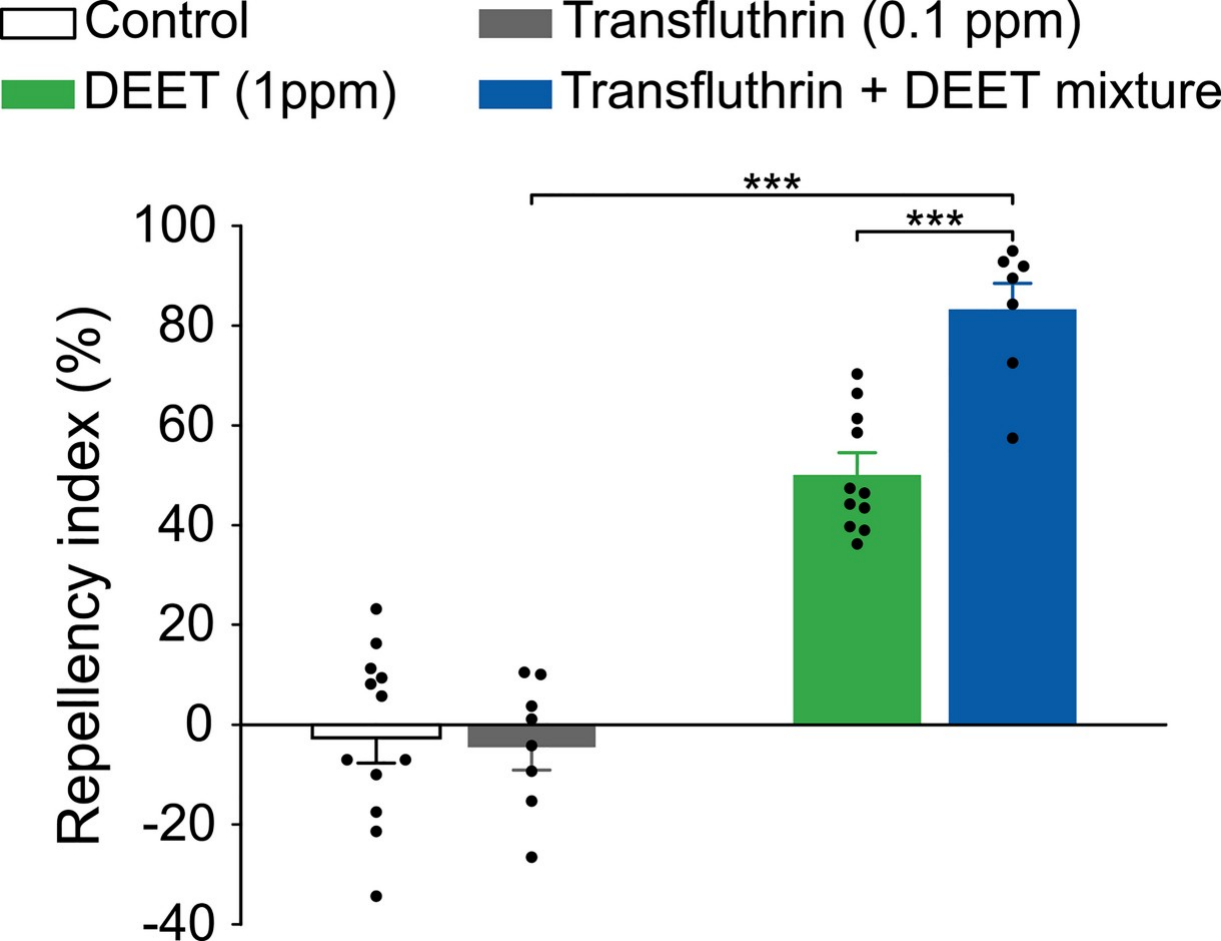
Transfluthrin potentiates the repellency by DEET in Rockefeller mosquitoes. Student’s *t*-test, for mixture *vs* transfluthrin: *t* = 12.82, *df* = 13, *P* < 0.001, for mixture *vs* DEET: *t* = 5.57, *df* = 16, *P* < 0.001, ***P < 0.001; *n* values for: mixture = 7, transfluthrin = 8, DEET = 11, and control = 12. The control represents the baseline activity in response to the solvent. Data are presented as mean ± SEM, and dots over the bars represent individual replicate values.

We were puzzled by the unexpected differences between our EAG results and those of Yang and colleagues [25]. One possibility, we hypothesized, is that the effect of transfluthrin on olfactory responses, as observed in our preliminary experiments and in the study of Yang and colleagues [25], may be caused in part by some type of cross-interaction between transfluthrin and additional olfactory response-eliciting compounds that are fortuitously present in certain transfluthrin samples. To test this hypothesis, we examined whether transfluthrin affects DEET repellency in Rockefeller mosquitoes. Remarkably, transfluthrin at 0.1 ppm (i.e., 2 ng/cm^2^), which did not elicit repellency alone, enhanced DEET repellency in Rockefeller mosquitoes (Fig 5).

### Commercial transfluthrin-based mosquito repellents possess both sodium channel-mediated and Or-mediated repellencies

**Fig 6:**
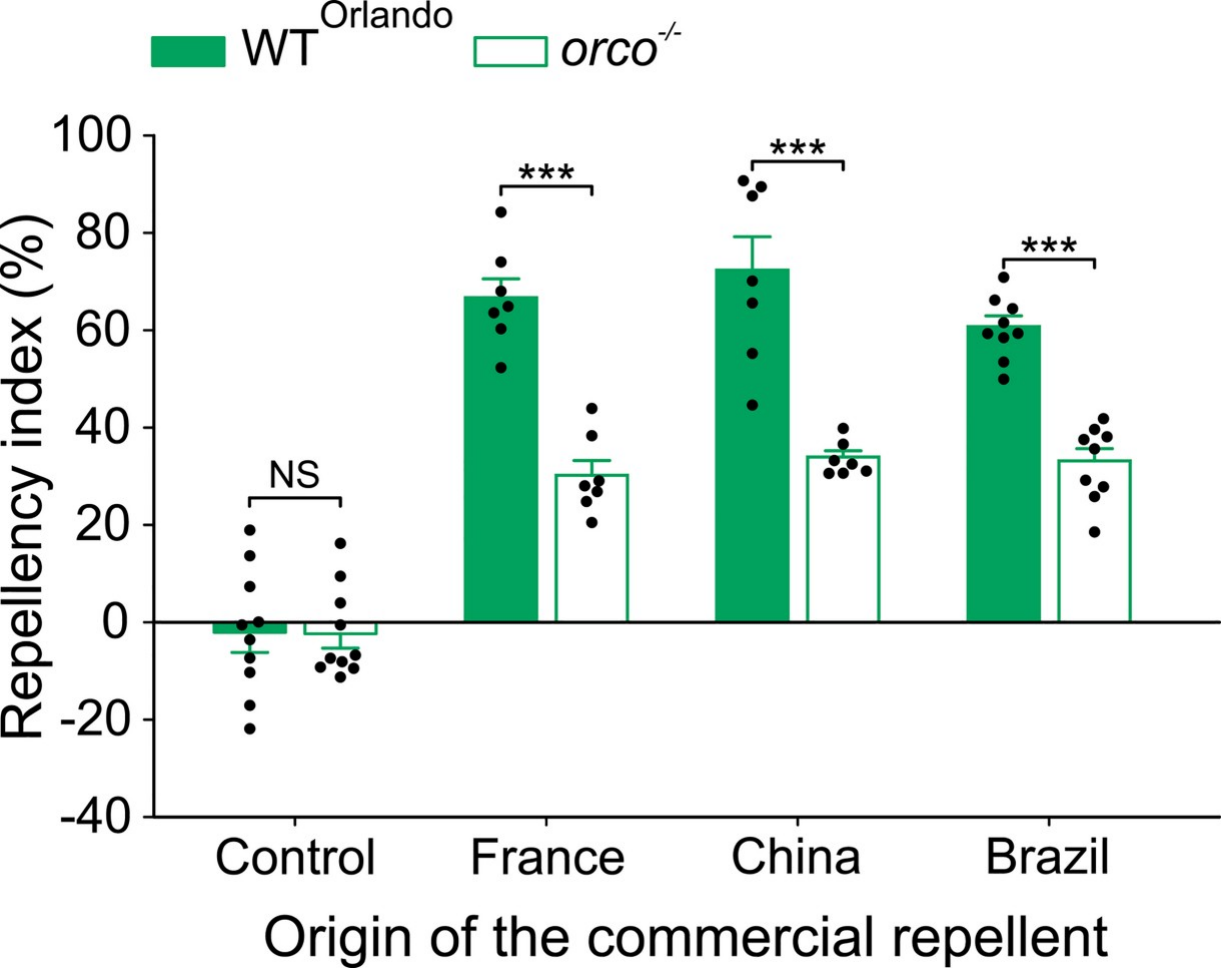
Repellency by commercial transfluthrin products consists of both Or-mediated and Or-independent repellency in *Ae*. *aegypti*. Dilutions were made in order to adjust the concentration of transfluthrin to 1 ppm, accordingly to the original concentration indicated in the product label. The Orlando and *orco*^*-/-*^ mosquito lines were used. Student’s *t*-test, for control: *t* = 0.05, *df* = 18, *P* = 0.963, for France: *t* = 7.40, *df* = 12, *P* < 0.001, for Brazil: *t* = 8.31, *df* = 16, *P* < 0.001, Mann-Whitney Rank Sum test, for China: *U* = 0.0, *P* < 0.001, NS = not significant, ***P < 0.001; *n* = 9 cages for Brazil in both lines, *n* = 10 cages for both controls, and *n* = 7 cages for the rest. The controls represent the baseline activity in response to the solvent. Data are presented as mean ± SEM. Each black circle over the bars represents individual replicate values.

It is possible that commercial transfluthrin repellent products may contain impurities that could activate Ors. To test that, we collected three commercial transfluthrin-based mosquito repellent products from Brazil, China, and France. The percentage of the active ingredient (i.e., transfluthrin) in these products ranged from 0.80 to 0.92%. We diluted each product to the concentration of 1 ppm of transfluthrin for direct comparison with the Sigma-Aldrich transfluthrin in the hand-in-cage assay. Remarkably, all three commercial transfluthrin products elicited repellency greater than that by Sigma-Aldrich’s transfluthrin in Orlando mosquitoes. Furthermore, while the repellency by the Sigma-Aldrich transfluthrin in *orco*^*-/-*^ mosquitoes remained intact (Fig 3), repellency by these products were significantly reduced in *orco*^*-/-*^ mosquitoes (Fig 6). Interestingly, the remaining percentage of repellency in *orco*^*-/-*^ mosquitoes by the commercial products was comparable to that by the Sigma-Aldrich transfluthrin in wild-type Rockefeller and Orlando mosquitoes (Figs 1B and 3). Together, these results suggest that all examined commercial transfluthrin products contain additional compounds that activate Ors, which contribute to repellency.

## Discussion

Current understanding on how insect repellents, such as DEET, evoke spatial repellency can be categorized into three major modes of action: activation of olfactory sensory receptors that mediate repellent pathways [29,38–42], inhibition of olfactory sensory processing of attraction cues from a host [24,43–45] and/or chemically masking attractants thereby reducing host attraction [38,42,46]. In this study, we provide experimental evidence for a new mechanism of spatial repellency *via* activation of sodium channels, independently of activation or inhibition of ORNs, as a principal mechanism of transfluthrin repellency. Together with recent demonstration of activation of a transient receptor potential cation channel (TrpA1) by nepetalactone as a basis of insect repellency [47], our study provides compelling evidence for an emerging mechanism of insect repellency via activation of insect ion channels other than Orco/Ors. Moreover, our discovery of potential synergism between sodium channel activating transfluthrin and Or-activating DEET points to an exciting possibility to use natural pyrethrins, synthetic pyrethroids or other sodium channel activators as general synergists to increase the potency of DEET and possibly other Or-activating insect repellents.

Repellency by insecticides that disrupt the function of the nervous system is often called irritancy, which occurs as a sublethal neurotoxic event and often at direct contact with treated surfaces [21,48,49]. Prior to this study, a common belief of the mechanism of pyrethroid repellency is that pyrethroids causes a general neurotoxic effect, which consequently disables mosquitoes from finding hosts [42]. This is an attractive hypothesis, especially when pyrethroid concentrations reach sublethal levels and mosquitoes are in direct contact and/or are very close to pyrethroid vapor-emitting devices. Transfluthrin at such sublethal concentrations would likely disrupt the normal function of electrical signaling by altering the gating of sodium channels, resulting in certain behavioral impairments, as observed in our behavioral assay for 100 ppm or higher. In this study, we provide experimental evidence showing that transfluthrin-mediated spatial repellency can occur at nonlethal concentrations (e.g., at 1 ppm) at which no behavioral impairment was observed during and after exposure. In a natural setting, mosquitoes would encounter such low concentrations of transfluthrin vapor first at a distance, before getting closer to the emanating source. Our results suggest that transfluthrin-mediated spatial repellency *via* activation of sodium channels already occurs before mosquitoes encounter higher transfluthrin concentrations.

Our results from the experiments using the inactive isomer and *Orco* mutant mosquitoes show that activation of sodium channels alone is sufficient for transfluthrin repellency. Reduced repellency in KDR:ROCK mosquitoes is most likely due to the presence of the two kdr mutations (S989P and V1016G) which reduce the action of transfluthrin on sodium channels. Possible genetic differences other than the *kdr* allele are possible, but still unknown between Rockefeller and ROCK:KDR strains (Smith et al., 2018). How activation of sodium channels alone evokes repellency remains to be further investigated. Differential sensitivity of insect sodium channel variants (generated *via* alternative splicing or RNA editing) to pyrethroids has been well-documented [5]. We predict that transfluthrin may activate hypersensitive sodium channel variants in certain neural circuits which control repellency or inhibit mosquito’s ability to find host. Activation of such a circuit(s) directly or indirectly potentiate neuronal excitability of specific Or-mediated repellent pathways. Functional identification and localization of transfluthrin–hypersensitive sodium channel variants will be necessary to advance further mechanistic understanding of the transfluthrin synergism in insect olfaction. Future research should also use direct imaging of neural circuits in the mosquito brain to gain further insights into how transfluthrin-mediated sodium channel-activation impacts olfactory signal processing in the insect brain.

Results from this study likely have immediate and significant practical implications. In particular, the synergism between sodium channel-activating pyrethroids and DEET as discovered in this study, could provide a basis for reciprocally augmenting the potency of two classes of insect repellents and lowering the concentration of each repellent used. This would be especially significant in terms of minimizing both, the costs and the toxic effects of transfluthrin and other repellent products, while maintaining adequate repellency and durability of commercial synthetic insect repellents. Fortuitously, current commercial transfluthrin repellent products may already exploited the hidden synergism in repellency by sodium channel-mediated transfluthrin and unknown Or-activating component(s) in these products. Future determination of these components that potentially synergize transfluthrin repellency may provide new lead compounds for the development of more potent insect repellent mixtures.

Reduced transfluthrin repellency against kdr mosquitoes as reported in our study and others’ studies [21,25] suggests potential reduction in the effectiveness of pyrethroid-based products in repelling pyrethroid resistant insect populations. Further studies are needed to investigate whether extensive use of volatile pyrethroids as spatial repellents influences the selection of pyrethroid resistance in natural populations. Better understanding of mosquito behaviors in response to volatile pyrethroids could provide valuable information for developing more judicious strategies to use this unique class of insecticides in the control of vector-borne human diseases. Our findings could also spur renewed interests in discovering new sodium channel activators with unique receptor sites on sodium channels that are distinct from the pyrethroid receptor sites [50,51]. Such new chemistries could be more effective in repel kdr mosquitoes. Overall, results from this study illustrate the first example of sodium channel activation-based repellent mechanism in insects, and provide a novel framework for future development of synergistic insect repellent mixtures to combat mosquitoes and other human disease vectors.

## Supporting information

S1 Table Odorants products used in the current study. (PDF)

S1 Text Supplementary methods. (PDF)

S1 Fig No electroantennogram signals by transfluthrin were detected from pyrethroid-resistant (A) and wild-type (B) mosquitoes.
(PDF)

S2 Fig Transfluthrin resistance of KDR:ROCK mosquitoes and lack of toxicity of the *1S-cis* isomer against transfluthrin susceptible Rockefeller mosquitoes. (PDF)

S1 Data Supporting data. (XLSX)
