# Supplementary material for: Sodium channel activation underlies transfluthrin repellency in Aedes aegypti
Source: PLoS Negl Trop Dis. 2021 Jul 8;15(7):e0009546. doi: 10.1371/journal.pntd.0009546 (PMC8266078; doi:10.1371/journal.pntd.0009546)
Supplement: S1 Table — (PDF) [file pntd.0009546.s001.pdf]

S1 Table. Odorants products used in the current study.

Name	CAS	Brand/manufacturer	Prod. code	Lot or Batch	Purity (%)
Transfluthrin Sample A	118712-89-3	Sigma Aldrich	46114	BCBV5909	99.2
Transfluthrin Sample B	118712-89-3	Jiangsu Yangnong Chemical Co.	-	-	98.5
Transfluthrin Sample C	118712-89-3	Sigma Aldrich	46114	BCBT5175	99.9
1S-cis isomer of transfluthrin	-	Jiangsu Yangnong Chemical Co.	-	-	97.75
DEET	134-62-3	Sigma Aldrich	36542	BCBS3631V	98.8
1-octen-3-ol	3391-86-4	Aldrich	O5284	MKBT3320V	99.4
Acetone	67-64-1	Sigma Aldrich	270725	SHBK1371	99.98
Dimethyl sulfoxide	67-68-5	Sigma	D2650	RNBF1057	100
L-(+)-Lactic acid	79-33-4	Sigma	L1750	067K0748	98
Liquid Repellent from Brazil	-	SBP/Reckitt Benckiser	-	ZOB2781O2T	0.8[a]
Liquid Repellent from China	-	Guard Sénce	-	-	0.9[a]
Liquid Repellent from France	-	Raid/Johnson&Johnson	-	-	0.9[a]

[a]Products composed of mixtures, with percentage indicated for the active ingredient (i.e., transfluthrin).
