# Supplementary material for: Sodium channel activation underlies transfluthrin repellency in Aedes aegypti
Source: PLoS Negl Trop Dis. 2021 Jul 8;15(7):e0009546. doi: 10.1371/journal.pntd.0009546 (PMC8266078; doi:10.1371/journal.pntd.0009546)
Supplement: S1 Text — (PDF) [file pntd.0009546.s002.pdf]

S1 Text. Supplementary methods

Hand-in-cage assay detailed set up.

The hand-in-cage assay set consisted of metal mosquito cages (30 cm x 30 cm x 30 cm) (BioQuip, Rancho Dominguea, CA) equipped with a digital camera (e-con Systems Inc, San Jose, CA, model: e-CAM51A) on its top and a human hand wearing a modified glove placed inside the cage (Fig 1A). The modified glove was made by creating a rectangle window (6 cm x 5 cm) on the back side of a nitrile rubber glove (Ansell Protective Products, Coshoton, OH, part number: 37-155). A rectangle magnetic frame that is slightly larger than the dimension of the window was glued onto the cut window, which served as a base for stacking additional magnetic window frames. One piece of test compound-treated polyester netting (Shason Textile Inc., part number: WS-B532-111, Walmart # 567948282, white; slightly larger than the dimension of the window, but smaller than the outer edge dimensions of the magnetic frames) was placed on this fixed magnetic frame, which was ~3.0 mm above the glove. The second piece of the netting was untreated and placed ~8.0 mm above the treated netting using a stack of four magnetic frames. The stacked magnetic frames were further secured with two binder clips. The stacking creates sufficient space between the treated netting and the untreated netting so that mosquitoes that land on the open window were not able to contact the treated netting or contact and pierce the skin of the hand in the glove. The hand makes no contact with the treated netting.

Twenty-four hours before the assay, four to nine days-old female mosquitoes (about 40, mated, non-blood fed) were transferred into each of 10 cages (i.e., 10

replicates). The cages were kept in an incubator (27 ºC, relative humidity of ~50% and photoperiod of 12h). A cotton ball soaked with distilled water was placed on the top of each cage. Just before each run of the hand-in-cage assay, a lab assistant transferred the prepared cage from the incubator to a bench in the assay room. In the meantime, in an adjacent room, one researcher (i.e., tester) treated a piece of netting with 500 µl acetone or test compound dissolved in acetone in a glass Petri dish. After letting acetone evaporate (~7 min), the researcher assembled and put on a modified glove. Both personnel avoided use of any hand lotions and cosmetic products and wore white lab coats and gloves. The hand in the modified glove was introduced into the cage to initiate the assay. The mosquitoes landing on the top netting were recorded for five minutes by the video camera. The average time for each run was 7-8 min. Ten cages were tested with acetone treated netting first, then in the same sequence, the same ten cages were tested using test compound-treated netting or acetone-treated netting as control. Thus, the time interval between the two runs for each cage was at least 1.5 hours, allowing the mosquitoes to fully recover and residual vapors from experiments to be ventilated out of the room. Any cage that gave a low landing number (i.e.. < 50% of the average landing compared with other cages) in the first run was not continued with the second run (< 1% of all the replicates in our study).

For each cage, the number of lands (individual mosquitoes were not tracked, lands number may include multiple landings by same individual mosquitoes) on the window from second to fifth minutes was counted from two 5 min videos from two runs. A landing was considered as so when a mosquito come to a total land, closing its wings completely. If a mosquito only hovered over the netting, or bounced off before a complete land, while

still beating its wings, it was not considered a landing. Also, several mosquitoes would reach the top netting walking from the edges of the magnetic frame, and so, those mosquitoes entering the evaluation window was also counted as “landings”. Repellency index was calculated using the following equation: Percentage repellency = [1 - (cumulative number of landings on the window of treatment / cumulative number of landings on the window of solvent treatment)] x 100). After the second run, mosquitoes were visually assessed for signs of intoxication, such as uncoordinated movements, or knockdown, before disposal.

After the assay, to remove any residual chemicals on the cages, each cage was rinsed with 99% ethanol (spray), distilled water (thorough rinse) and then a second ethanol spray, before being left to air dry. The modified glove and its magnetic frames were soaked in ethanol (99%) in a container, then rinsed with distilled water and a second ethanol rinse, before being left to air dry.

Electroantennogram (EAG).

Electroantennogram was performed with 4-6 days old female mosquitoes. Both ground and recording electrodes were made from a glass capillary (Kimble Glass Inc. Kimax-51, Part. Nº = 34502 99, size = 0.8 -1.10 x 100 mm) pulled in a flaming/brown micropipette puller (Shutter Instrument Co. Model P-87, set to = Pull: 95, Vel: 150, Time: 155, Heat: ramp temperature + 7). Each electrode was filled with filtered standard EAG recording solution [0.1N KCl + 0.5% polyvinylpyrrolidone (PVP)]. The recording electrode consists of a glass electrode and a chlorinated silver wire (Beantown Chemical, Part. Nº = 140740-5M; 0.25 mm of diameter) inside the glass electrode. Just prior the recording,

the head of a female mosquito was excised with fine forceps and a small fraction of the right (looking from the top) antenna tip was cut off using a micro-dissecting precision blade. The ground electrode was introduced into the brain, from the soft membrane in the back portion of the head, and the whole set (i.e., electrode holder, electrode, head/antenna) was fixed to a hand micromanipulator (Märzhäuser Wetzlar Gmbh & Co. Model: MM33) to be visualized under a microscope. The tip of the antenna was placed inside the tip of the recording electrode with aid of a motorized micromanipulator (Burleigh PCS-6000, CA). The recording electrode were connected to the recoding set (as described in main text) and a 10 s signal was recorded for each odorant stimulation. The recordings were set to start 2 s before odor exposure, using EAG filter of 0.1 Hz, the smallest low-cutoff available. The purified and humidified air was delivered to the preparation through a glass tube (10-mm inner diameter) perforated by a small hole 10 cm away from the end of the tube, into which the tip of the odorant cartridge, could be inserted.

In EAG assays it is normally applied 10 µl of odorant solution per pipet cartridge at the 10^{-1} or lower dilutions [1–4]. However, dimethyl sulfoxide (DMSO) elicited small EAG signals. Therefore, a potential small signal from transfluthrin could be overlapped with this solvent induced signal. Thus, we tested transfluthrin and other odorants without solvent (1 µl of pure compound corresponds to 10 µl of 10^{-1} solution when solvent-diluted) (Fig 2 and S1 Fig). A blank empty pipet cartridge was used as control.

Toxicological assay.

The toxicities of transfluthrin and *1S-cis* isomer against female mosquitoes were evaluated using a vapor toxicity assay. We placed twenty 5-day old mosquitoes in a 200 ml plastic cup and then covered the cup with a thin Parafilm “M” (Bemis, Neenah, WI). A vapor cartridge was made from a glass Pasteur pipette containing a piece of filter paper (4 x 40 mm) impregnated with 10 μl of tested compound solution in DMSO. To inject a controlled amount of compound vapor into the cup, the cartridge was connected to a pulse flow stimulus controller system (CS-55, Syntech), and its tip inserted into the covered cup. The cartridge was quickly warmed with a lighter for 10 s immediately followed by a 3 s pulse at 0.5 l/min. A vapor cartridge with DMSO was used as control. The cartridge warming-up step was used to generate more vapor which allowed us to observe knockdown of KDR:ROCK mosquitoes. One hour after vapor exposure, the mosquitoes in the cup were released into a mosquito cage (30 cm x 30 cm x 30 cm) (BioQuip) and the number of mosquitoes that were on their back were counted. Five replicates for each of 6 concentrations were carried out. Data was analyzed by the PROBIT procedure in the SAS statistical software package.

References

1. Den Otter CJ, Behan M, Maes FW. Single cell responses in female *Pieris brassicae* (Lepidoptera: Pieridae) to plant volatiles and conspecific egg odours. J Insect Physiol. 1980;26(7):465–72.

2. Pelletier J, Guidolin A, Syed Z, Cornel AJ, Leal WS. Knockdown of a mosquito odorant-binding protein involved in the sensitive detection of oviposition attractants. J Chem Ecol. 2010;36(3):245–8.

3. Stanczyk NM, Brookfield JFY, Ignell R, Logan JG, Field LM. Behavioral insensitivity to DEET in *Aedes aegypti* is a genetically determined trait residing in changes in sensillum function. Proc Natl Acad Sci U S A. 2010;107(19):8575–80.

4. Xu P, Choo YM, De La Rosa A, Leal WS. Mosquito odorant receptor for DEET and methyl jasmonate. Proc Natl Acad Sci U S A. 2014;111(46):16592–7.
