# Supplementary material for: Sodium channel activation underlies transfluthrin repellency in Aedes aegypti
Source: PLoS Negl Trop Dis. 2021 Jul 8;15(7):e0009546. doi: 10.1371/journal.pntd.0009546 (PMC8266078; doi:10.1371/journal.pntd.0009546)
Supplement: S1 Fig — No electroantennogram signals by transfluthrin were detected from pyrethroid-resistant (A) and wild-type (B) mosquitoes. (PDF) [file pntd.0009546.s003.pdf]

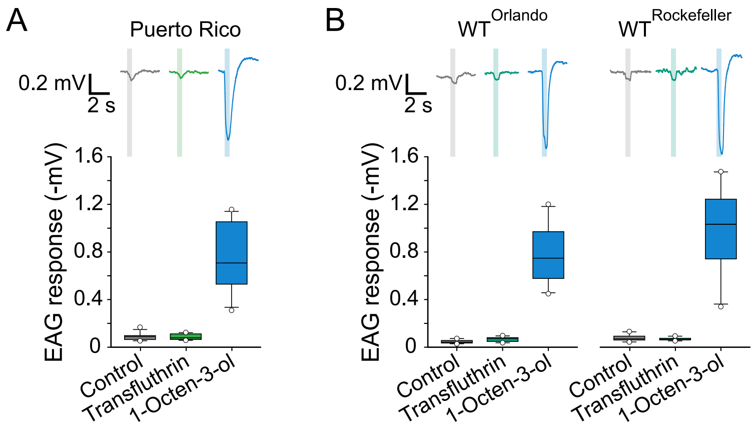

S1 Fig. No electroantennogram signals by transfluthrin were detected from pyrethroid-resistant (A) and wild-type (B) mosquitoes.

(A) Transfluthrin used was from Sigma (Lot: BCBT5175), and mosquito line was Puerto Rico. *n* = 12 antennae. (B) Transfluthrin used was from Jiangsu Yangnong Chemical Co. Ltd. (Jiangsu, China; 98.5% purity) and mosquito lines were both Orlando and Rockefeller; *n* = 10 antennae each mosquito line. 1-Octen-3-ol was applied as positive control following transfluthrin on the same antennae. In both panels representative traces are shown above each plot.
