# Supplementary material for: Sodium channel activation underlies transfluthrin repellency in Aedes aegypti
Source: PLoS Negl Trop Dis. 2021 Jul 8;15(7):e0009546. doi: 10.1371/journal.pntd.0009546 (PMC8266078; doi:10.1371/journal.pntd.0009546)
Supplement: S2 Fig — (PDF) [file pntd.0009546.s004.pdf]

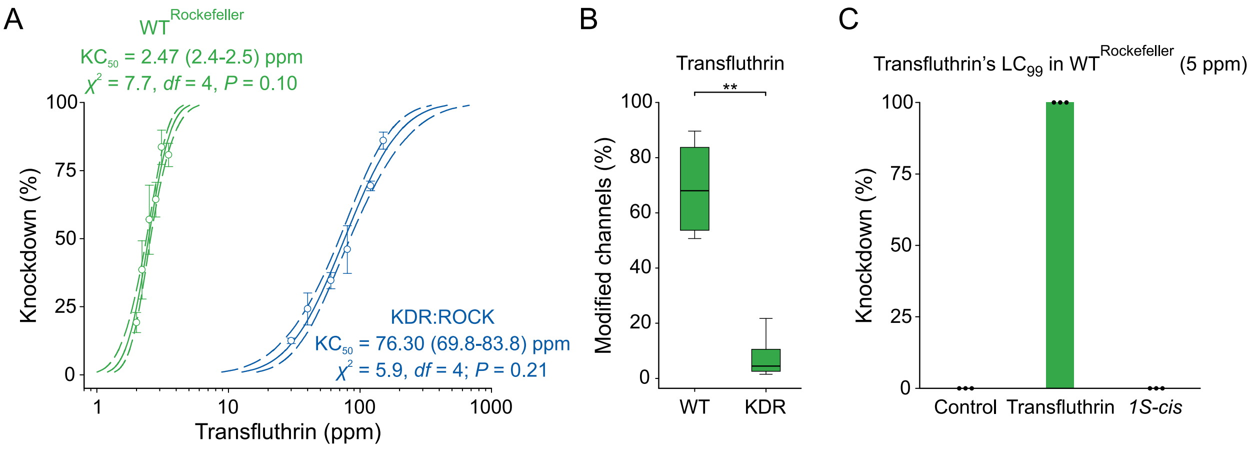

S2 Fig. Transfluthrin resistance of KDR:ROCK mosquitoes and lack of toxicity of the *1S-cis* isomer against transfluthrin susceptible Rockefeller mosquitoes.

(A) Concentration-dependent knockdown by transfluthrin vapor in Rockefeller and KDR:ROCK lines; values of parameters showed in the insets from the probit analysis; the solid line represents the curve estimate while the dashed lines represent both the lower and upper 95% confidence intervals; each circle represents the observed knockdown (mean ± SEM, from n = 5 replicates of 20 insects each). (B) Sensitivity of AaNa$_v$1-1 and mutant channels expressed in *Xenopus* oocytes to transfluthrin; The percentage of modified channels was estimated from two electrode voltage clamp tail current recordings as described in Fig 4B, with the equation: $M = [I_{tail} / (E_h - E_{Na})] / [I_{Na} / (E_t - E_{Na})] \times 100$; where I_{tail} is the maximal tail current amplitude, E_h is the potential to which the membrane is repolarized, E_{Na} is the reversal potential for sodium currents determined from the current-voltage curve, I_{Na} is the amplitude of the peak current during depolarization before transfluthrin exposure, and E_t is the potential of the step depolarization. Larger percentage of modified channels indicate a more sensitive channels as a smaller percentage indicates a less sensitive channel. Mann-Whitney Rank Sum test, $U = 0$, P =

0.002, ***P* < 0.01, *n* = 5 oocytes for $AaNa_v1$-1, and *n* = 8 oocytes for mutant channels. (C) No knockdown effect of *1S-cis* isomer on Rockefeller mosquitoes at 5 ppm which is the KC_{99} of transfluthrin for Rockefeller mosquitoes from panel A; *n* = 3 groups of 20 mosquitoes each.
